# Supplementary material for: Detailed molecular and epigenetic characterization of the pig IPEC-J2 and chicken SL-29 cell lines
Source: iScience. 2023 Feb 20;26(3):106252. doi: 10.1016/j.isci.2023.106252 (PMC10018572; doi:10.1016/j.isci.2023.106252)
Supplement: Data S1. Complete homer output for identified motifs in Pig IPECJ-2, related to Table 2 — Homer motif analysis results for histone modifications H3K4me1, H3K4me3, H3K27ac, and enhancer elements of pig IPECJ2 cell line. P-values >1e-10 are possible false positives. Within each folder (e.g. peak_files_CTCF) are the html files showing the identified motifs when using homer (e.g. homerResults.html). [file mmc2.zip › S5/Pig_IPECJ_2/peak_files_H3K4me3/homerResults.html]

peak\_files\_H3K4me3/ - Homer de novo Motif Results


# Homer *de novo* Motif Results (peak\_files\_H3K4me3/)

Known Motif Enrichment Results  
Gene Ontology Enrichment Results  
If Homer is having trouble matching a motif to a known motif, try copy/pasting the matrix file into
STAMP  
More information on motif finding results: HOMER
| Description of Results
| Tips
  
Total target sequences = 22090  
Total background sequences = 26339  
\* - possible false positive  

|  |  |  |  |  |  |  |  |  |
| --- | --- | --- | --- | --- | --- | --- | --- | --- |
| Rank | Motif | P-value | log P-pvalue | % of Targets | % of Background | STD(Bg STD) | Best Match/Details | Motif File |
| 1 | T C G A C G T A G C T A G C A T G C A T A T G C T A G C A T C G | 1e-316 | -7.278e+02 | 54.95% | 42.20% | 86.9bp (104.0bp) | PB0075.1\_Sp100\_1/Jaspar(0.662) More Information \| Similar Motifs Found | motif file (matrix) |
| 2 | T C G A T C G A G C T A T G A C A G T C A C T G T G C A C T G A C T G A A T G C | 1e-287 | -6.616e+02 | 4.45% | 1.09% | 85.1bp (101.2bp) | IRF2(IRF)/Erythroblas-IRF2-ChIP-Seq(GSE36985)/Homer(0.919) More Information \| Similar Motifs Found | motif file (matrix) |
| 3 | A G C T T C G A G A T C A C G T A G C T C G T A G A T C G T A C | 1e-220 | -5.067e+02 | 68.60% | 58.24% | 84.8bp (94.4bp) | SD0001.1\_at\_AC\_acceptor/Jaspar(0.855) More Information \| Similar Motifs Found | motif file (matrix) |
| 4 | C G A T T C G A C T G A A G C T A T G C T A C G G T A C C A T G | 1e-158 | -3.649e+02 | 52.62% | 43.61% | 86.1bp (104.1bp) | PB0185.1\_Tcf1\_2/Jaspar(0.745) More Information \| Similar Motifs Found | motif file (matrix) |
| 5 | T A G C A T C G A C T G T A C G T C G A T C G A C G T A T G C A | 1e-154 | -3.561e+02 | 63.08% | 54.25% | 85.6bp (101.0bp) | E2F(E2F)/Hela-CellCycle-Expression/Homer(0.763) More Information \| Similar Motifs Found | motif file (matrix) |
| 6 | A G T C A C G T A G T C A T G C A T C G T A C G T C G A G T C A G A C T G A T C | 1e-142 | -3.273e+02 | 40.71% | 32.55% | 84.3bp (102.3bp) | Elk4(ETS)/Hela-Elk4-ChIP-Seq(GSE31477)/Homer(0.770) More Information \| Similar Motifs Found | motif file (matrix) |
| 7 | G C T A T C G A T G A C A T G C A C T G A C G T G C A T G A T C | 1e-135 | -3.109e+02 | 42.47% | 34.42% | 85.2bp (100.3bp) | PB0046.1\_Mybl1\_1/Jaspar(0.816) More Information \| Similar Motifs Found | motif file (matrix) |
| 8 | A T C G A T C G A C G T G C A T A T C G A T G C T A C G T C G A T C G A T C G A | 1e-134 | -3.095e+02 | 45.51% | 37.36% | 85.2bp (97.1bp) | IRF4(IRF)/GM12878-IRF4-ChIP-Seq(GSE32465)/Homer(0.681) More Information \| Similar Motifs Found | motif file (matrix) |
| 9 | T G A C A T G C T A C G T G A C C T A G A C T G G C A T C G A T | 1e-102 | -2.352e+02 | 26.47% | 20.44% | 84.7bp (106.7bp) | MYB(HTH)/ERMYB-Myb-ChIPSeq(GSE22095)/Homer(0.768) More Information \| Similar Motifs Found | motif file (matrix) |
| 10 | C T G A T C G A T G C A T C G A C T A G A T C G T A G C A T C G T A G C A T C G | 1e-96 | -2.216e+02 | 58.62% | 51.63% | 85.3bp (103.6bp) | PB0008.1\_E2F2\_1/Jaspar(0.788) More Information \| Similar Motifs Found | motif file (matrix) |
| 11 | T A G C C A T G T A G C C A G T C G T A G T A C T A G C T G A C | 1e-92 | -2.122e+02 | 40.98% | 34.36% | 83.4bp (104.8bp) | PB0156.1\_Plagl1\_2/Jaspar(0.672) More Information \| Similar Motifs Found | motif file (matrix) |
| 12 | C G A T G C T A T G A C G T A C A G C T G C A T T G A C T A C G | 1e-90 | -2.080e+02 | 26.21% | 20.54% | 85.9bp (98.8bp) | SD0001.1\_at\_AC\_acceptor/Jaspar(0.641) More Information \| Similar Motifs Found | motif file (matrix) |
| 13 | T A C G T A C G G C T A C G T A G C A T G C A T G T A C T A C G A C T G G A C T T C A G A C G T T C G A | 1e-71 | -1.638e+02 | 44.23% | 38.33% | 83.9bp (96.4bp) | PAX4/MA0068.2/Jaspar(0.657) More Information \| Similar Motifs Found | motif file (matrix) |
| 14 | T G A C A C T G A G C T A G C T G C A T C G T A C T G A T G C A | 1e-65 | -1.507e+02 | 17.92% | 13.79% | 84.4bp (100.1bp) | BARHL1/MA0877.2/Jaspar(0.791) More Information \| Similar Motifs Found | motif file (matrix) |
| 15 | A T C G T A G C C A G T G T C A T A C G A T G C C A G T G T C A | 1e-60 | -1.386e+02 | 25.31% | 20.72% | 88.1bp (100.4bp) | PB0154.1\_Osr1\_2/Jaspar(0.636) More Information \| Similar Motifs Found | motif file (matrix) |
| 16 | T A C G G T A C G C A T C T G A C G T A T A G C G C A T A T G C | 1e-53 | -1.242e+02 | 31.50% | 26.79% | 86.9bp (92.1bp) | MafA(bZIP)/Islet-MafA-ChIP-Seq(GSE30298)/Homer(0.714) More Information \| Similar Motifs Found | motif file (matrix) |
| 17 | A G T C A C T G G T C A A G T C C G T A A C G T A C T G A G T C | 1e-53 | -1.229e+02 | 5.25% | 3.25% | 85.9bp (101.7bp) | MXI1/MA1108.2/Jaspar(0.702) More Information \| Similar Motifs Found | motif file (matrix) |
| 18 | A G T C A C G T A C G T C G T A A G T C A G T C A C G T A C T G A C T G A G T C A G T C A C T G A C T G | 1e-42 | -9.827e+01 | 59.30% | 54.70% | 82.4bp (95.7bp) | SD0001.1\_at\_AC\_acceptor/Jaspar(0.704) More Information \| Similar Motifs Found | motif file (matrix) |
| 19 | T A G C T A C G T C G A T A G C A G T C A T G C A C G T G C T A A C G T A G T C | 1e-40 | -9.362e+01 | 13.68% | 10.78% | 81.7bp (95.9bp) | LRF(Zf)/Erythroblasts-ZBTB7A-ChIP-Seq(GSE74977)/Homer(0.660) More Information \| Similar Motifs Found | motif file (matrix) |
| 20 | T C A G T A G C C G A T T G A C C T A G C T A G G A C T T C G A G C T A C G T A | 1e-32 | -7.546e+01 | 6.23% | 4.46% | 86.5bp (96.0bp) | HOXC13/MA0907.1/Jaspar(0.629) More Information \| Similar Motifs Found | motif file (matrix) |
| 21 | A T C G A C G T T A G C A T C G T C A G T G C A T A G C A T C G A T G C G A C T | 1e-31 | -7.175e+01 | 25.98% | 22.63% | 83.7bp (101.6bp) | HINFP/MA0131.2/Jaspar(0.660) More Information \| Similar Motifs Found | motif file (matrix) |
| 22 | A C T G G A T C G T C A C T A G A G T C G T C A A C T G A G T C | 1e-28 | -6.557e+01 | 22.74% | 19.70% | 84.7bp (98.5bp) | NHLH1/MA0048.2/Jaspar(0.752) More Information \| Similar Motifs Found | motif file (matrix) |
| 23 | A T G C A C T G C G T A C G T A A C T G A T G C A G T C A C T G C G T A C T G A A C T G C T A G T A C G | 1e-27 | -6.248e+01 | 0.16% | 0.01% | 76.0bp (65.6bp) | POL008.1\_DCE\_S\_I/Jaspar(0.634) More Information \| Similar Motifs Found | motif file (matrix) |
| 24 | A T G C A G T C C A T G A C G T A G T C C G T A A G T C A G T C C T G A A C G T A T C G A C T G A C G T | 1e-26 | -6.093e+01 | 0.14% | 0.01% | 81.8bp (57.1bp) | Rfx1(HTH)/NPC-H3K4me1-ChIP-Seq(GSE16256)/Homer(0.626) More Information \| Similar Motifs Found | motif file (matrix) |
| 25 | A C T G T C G A C T G A C T G A C G T A C T A G G T A C A G T C A C G T A G T C A T G C A C T G A T C G | 1e-25 | -5.804e+01 | 0.13% | 0.01% | 82.0bp (60.5bp) | ZNF341/MA1655.1/Jaspar(0.625) More Information \| Similar Motifs Found | motif file (matrix) |
| 26 | C G T A C A T G A C T G C G T A G T C A T A G C A G T C A C G T C G T A C G T A C T G A A G T C A C G T | 1e-24 | -5.658e+01 | 0.10% | 0.01% | 82.4bp (0.0bp) | IRF6/MA1509.1/Jaspar(0.685) More Information \| Similar Motifs Found | motif file (matrix) |
| 27 | A C G T A G T C C A G T A T C G G T C A C A G T A G C T A G T C G C A T A T C G T C G A C A T G G T A C | 1e-20 | -4.682e+01 | 0.11% | 0.01% | 84.3bp (64.4bp) | PRDM4/MA1647.1/Jaspar(0.605) More Information \| Similar Motifs Found | motif file (matrix) |
| 28 | G T A C G A C T A C G T A G C T A G T C C T G A T A C G C G T A G T C A A C G T T G C A G T C A C G T A | 1e-20 | -4.682e+01 | 0.11% | 0.01% | 86.0bp (17.1bp) | IRF:BATF(IRF:bZIP)/pDC-Irf8-ChIP-Seq(GSE66899)/Homer(0.669) More Information \| Similar Motifs Found | motif file (matrix) |
| 29 | G C T A T A C G G T C A G A T C T C G A A G T C C A T G C A T G C T G A T A C G C T G A T C G A C T G A | 1e-19 | -4.398e+01 | 0.14% | 0.02% | 84.5bp (49.1bp) | PRDM1/MA0508.3/Jaspar(0.597) More Information \| Similar Motifs Found | motif file (matrix) |
| 30 | A T G C A T G C A T C G T G A C C T G A T A C G T A G C A C G T C G T A A C G T A C T G C T A G T A C G | 1e-16 | -3.738e+01 | 0.13% | 0.02% | 84.4bp (52.1bp) | NHLH1/MA0048.2/Jaspar(0.630) More Information \| Similar Motifs Found | motif file (matrix) |
| 31 | A T G C A T G C T A C G A T G C T A G C G T C A A G C T A G C T A G T C G A C T A G T C G T A C A C T G | 1e-16 | -3.738e+01 | 0.13% | 0.02% | 86.6bp (59.8bp) | YY2/MA0748.2/Jaspar(0.708) More Information \| Similar Motifs Found | motif file (matrix) |
| 32 | A G T C G T A C C A T G G A T C G A T C A G C T A G T C G T A C T C G A T G C A T C G A C A T G T C G A | 1e-14 | -3.387e+01 | 0.13% | 0.02% | 89.0bp (47.2bp) | ZNF460/MA1596.1/Jaspar(0.682) More Information \| Similar Motifs Found | motif file (matrix) |
| 33 | A C T G A C G T A G T C A G T C A G T C A C T G A C T G C G T A A C G T A G T C A G T C A C T G A C T G | 1e-14 | -3.386e+01 | 2.65% | 1.89% | 83.5bp (97.7bp) | PB0077.1\_Spdef\_1/Jaspar(0.605) More Information \| Similar Motifs Found | motif file (matrix) |
| 34 | C G A T C G T A A G C T A G T C A C T G A T C G A C T G A C T G | 1e-14 | -3.267e+01 | 1.41% | 0.88% | 84.7bp (59.2bp) | PB0128.1\_Gcm1\_2/Jaspar(0.709) More Information \| Similar Motifs Found | motif file (matrix) |
| 35 \* | C G T A A G T C A C G T A G T C A G T C A C T G A G T C C G T A A C T G A G T C A C G T A G T C A C T G | 1e-8 | -1.866e+01 | 0.13% | 0.04% | 66.9bp (36.5bp) | Bach1::Mafk/MA0591.1/Jaspar(0.566) More Information \| Similar Motifs Found | motif file (matrix) |
